# The Effects of Protein Nutrition on Muscle Function in Critical Illness: A Systematic Review and Meta-Analysis

**DOI:** 10.3390/nu17162613

**Published:** 2025-08-12

**Authors:** Mohamed A. Mohamed, Brett Doleman, Bethan E. Phillips, John P Williams

**Affiliations:** 1 Centre of Metabolism, Ageing and Physiology (COMAP), MRC-Versus Arthritis Centre of Musculoskeletal Ageing Research (CMAR) and Nottingham NIHR Biomedical Research Centre, School of Medicine, University of Nottingham, Derby DE22 3DT, UK; mohamed.mohamed4@nottingham.ac.uk (M.A.M.); brett.doleman2@nottingham.ac.uk (B.D.); beth.phillips@nottingham.ac.uk (B.E.P.); 2 Department of Anaesthetics and Intensive Care, Royal Derby Hospital, Derby DE22 3NE, UK

**Keywords:** protein nutrition, critical illness, muscle strength

## Abstract

Background: owing to altered protein metabolism during critical illness, skeletal muscles are utilised as a source of protein, with subsequent debilitating effects on both muscle structure and function. Protein nutrition has been shown to improve clinical outcomes in critically unwell patients; however, the impact on muscle function is less established. Therefore, the aim of this review was to systematically determine the effect of protein dose on skeletal muscle strength in critically ill patients. Methods: we searched five databases (Ovid MEDLINE, Embase, Emcare, CINAHL, and PubMed) and clinical trial registers for randomised controlled trials (RCTs) of non-pregnant, adult patients admitted to an intensive care unit (ICU), which assessed the impact of different doses of protein nutrition on muscle strength. Studies investigating only muscle structure or with co-interventions were excluded. Six RCTs were eligible for inclusion, and five were suitable for meta-analysis. Results: there was a significant difference in skeletal muscle strength with higher versus lower protein intakes, with a mean difference of 2.36 kg (95% CI: 0.37–4.35). The mean difference in protein dose was 0.46 g/kg/d (95% CI: 0.29–0.64). Inconsistency was evident across the included studies, with risk of bias ranging from moderate to high. Conclusion: muscle strength of ICU patients does appear to be affected by different protein doses. However, trials focusing on muscle function are limited by number and quality, highlighting a clear need for future work.

## 1. Introduction

Over 130,000 patients are admitted to an intensive care unit (ICU) each year in the UK alone, which, when scaled to the US population, would equate to 650,000 admissions per year [1]. Muscle wasting, known as ICU Acquired Weakness (ICUAW), is a debilitating outcome in critical illness [2], and recognition of this has led to increased clinical and research activity in this space [3]. Exemplifying the rapid decline in muscle ‘health’ (i.e., mass and function) in critical illness, muscle loss has been observed as early as one week post-ICU admission [2], with the greatest rates of (lower limb) atrophy within the first few days of admission. Whilst withdrawal of contractile stimuli, a key anabolic stimulus [4], is clearly a component of muscle wasting in critical illness, rates of atrophy are far greater in this setting than in non-clinical experimental models of disuse [5], highlighting that ICU-acquired muscle wasting is both complex and multifactorial. While it principally arises from elevated muscle protein catabolism and concomitant blunted anabolism [6], inflammation, malnutrition, and pre-existing chronic disease, including sarcopenia [7], all contribute to ICUAW [8], which is implicated in poor outcome states, including lengthy ICU stay, decreased ventilator-free days, and worsened mortality rate [9].

With significant alterations in protein metabolism seen in ICU patients [2], many researchers have focused on the potential role of nutritional interventions to improve clinical outcomes in these patients [10,11]. During intensive tissue stress (e.g., tissue injury or sepsis), protein metabolism alters to a state of accelerated skeletal muscle catabolism as amino acids are released into the circulation to be used for hepatic gluconeogenesis and synthesis of acute-phase reactants and immune cells [12]. Providing higher doses of protein to support this catabolic state has been shown to have benefits for critically unwell patients, both with and without sepsis, with expert consensus [13] and experimental evidence advocating protein provision of 1.2–1.5 g/kg/d to improve outcomes such as 60-day mortality and ventilator-free days [14]. In addition, researchers have also demonstrated that a protein intake of less than 0.8 g/kg/d is associated with worse clinical outcomes, prolonged hospital stay, and consequent economic burden [15]. Concerns around the safety of high protein in ICU patients cannot, however, be ignored, and although high protein supply (2 g/kg/d) has been shown to be safe and feasible by some [16], which was asserted by Preiser, who also pointed out that protein synthesis increased in response to high nitrogen provision at the early stage of critical illness [17], the recent international multi-centre EFFORT trial reported that high (>2.2 g/kg/d) compared to low (<1.2 g/kg/d) protein provision did not improve time to discharge, and may have resulted in worse clinical outcomes for those with acute kidney injury or high organ failure scores [18]. Beyond potential clinical negative sequelae of high protein doses, further cellular adaptive consequences were highlighted in the acute phase of critical illness, including auto-cannibalism (resistance to anabolic stimuli to direct amino acids to supply vital organs via gluconeogenesis) [19]. Although it is beyond the scope of this review, there is a current focus in the literature on providing adequate protein supplements as an adjuvant along with physical rehabilitation in a multidisciplinary approach for ICUAW [20].

Despite clear evidence that preserved muscle mass and function are associated with improved health-related quality of life, including in those recovering from a period in the ICU [21], to date, the majority of nutritional studies, aside from those that have explored clinical outcomes, have focused on the preservation of muscle mass and/or structure [10] rather than muscle function. Furthermore, one review that did explore the impact of different nutritional interventions on muscle function did not focus on protein nutrition, nor did it include any studies that reported interventions altering the route, timing, speed, amount, or composition of nutritional delivery [22]. The main focus of this review, unlike many previously published, is skeletal muscle function and not structure. While we appreciate the strong relationship between these aspects in many situations, we are also aware of disparities in losses (i.e., as seen in sarcopenia) [23].

One potential explanation for the lack of studies assessing muscle function in ICU patients is the practical difficulty in assessing this (i.e., compared to ultrasound to determine muscle mass). However, while it is recognised that a large proportion of ICU patients are not able to complete many of the established and well-validated measures of physical function (e.g., Short Physical Performance Battery (SPPB) [24] or Timed Up-And-Go [25]), handgrip dynamometry is a straightforward test to assess muscle strength, which has been proven feasible for use in the ICU setting [26]. Moreover, handgrip strength (HGS) has been shown across numerous patient populations (including ICU [27]) to be associated with various other aspects of muscle function [28] and has been reported as an independent risk factor for in-hospital mortality [27].

This systematic review aims to compare the effect of low versus high protein provision on skeletal muscle function in critically ill patients. To our knowledge, this is the first review to focus on the sole effect of different protein doses, excluding the confounding effect of variable caloric intake.

## 2. Methods

We used the 2020 Preferred Reporting Items for Systematic Reviews and Meta-Analyses (PRISMA) statement and updated Cochrane handbook guidelines [29] to construct this systematic review [30]. This review has been registered with PROSPERO under the number CRD42022320190.

### 2.1. Eligibility Criteria

As the gold standard of evidence, only randomised controlled trials (RCTs) were included. These studies could be published as journal articles, unpublished manuscripts, or conference abstracts, and no date restrictions were employed. Study populations of interest were adults (≥18 years) admitted to the ICU. High-dose protein supplementation was defined by the publication via any route of delivery (e.g., enteral, total parenteral, and supplemental parenteral nutrition). Only studies aiming for equal energy delivery (prespecified isocaloric target) between intervention and control groups were included. Studies were included if they reported muscle strength as a pre-determined outcome. Studies were excluded if protein supplementation was part of a wider package of intervention (e.g., with adjuvant exercise), as were those that only assessed muscle architecture (i.e., muscle mass and not function).

### 2.2. Information Sources and Search Strategy

Facilitated by an experienced librarian, a systematic search of five databases (MEDLINE, Emcare, Embase, PubMed, and CINAHL) was conducted using MeSH terms. In addition, a study registry (ClinicalTrials.gov) was searched for ongoing studies. Snowball searching was conducted via reference lists of eligible trials. The full search strategy can be seen in Supplementary Material S1.

### 2.3. Data Collection and Presentation

Data were extracted from records by two reviewers independently (MM and BD); discrepancies were referred to JPW. Due to limited data being presented in the included studies, it was not possible to report a change in outcomes over the study intervention periods. Therefore, only final values after the intervention period were reported. Two studies [31,32] reported medians and interquartile ranges for continuous outcomes, so means and standard deviations were calculated using Wan’s method. Where results were presented only in a graphical format or the number of participants was not clear, online graphical analysis software was used “http://plotdigitizer.com” (accessed on 16 June 2025), and study investigators were contacted to obtain accurate numerical values. Where results were reported for subgroups, an online formula was used to gather data into one intervention and one control group “http://www.obg.cuhk.edu.hk”(accessed on 3 March 2025).

Outcomes are presented as mean differences (MDs) with 95% confidence intervals (CIs) using a random-effects model (restricted maximum likelihood) given its reliability in estimating between-study variance.

### 2.4. Methodology of Risk of Bias Assessment

The revised Cochrane risk of bias tool for RCTs (RoB 2.0) was used by two authors (MM and BD) independently to critically appraise the included studies [15,31,32,33,34,35]. RoB 2.0 addresses 5 domains, as follows: (1) bias arising from randomization; (2) bias due to deviation from intended intervention; (3) bias due to missing data; (4) bias in measurement of the outcome; and (5) bias in selection of the reported results, with each domain judged as low risk, high risk, or some concerns. Thereafter, an overall appraisal of the study was conducted using the highest risk of bias in any of the mentioned domains. Supporting notes were added to justify the assessment of each domain. Any disagreement between the two reviewers was resolved by discussion with JPW.

### 2.5. Effect Measures and Synthesis Methods

All analysis was conducted using “RevMan software 5.4.1”. Data were aggregated using generic inverse variance. Skeletal muscle strength was analysed as the functional continuous outcome of interest. A random-effects model was used due to heterogeneity between the studies, including different protein doses and different time points at which the outcome was assessed. Statistical heterogeneity was calculated using the Tau-squared and I-squared statistics, and we followed Cochrane guidelines to assess the degree of heterogeneity (i.e., 0% to 40%: not significant, 30% to 60%: moderate heterogeneity, 50% to 90%: substantial heterogeneity, and 75% to 100%: considerable heterogeneity). A forest plot was created to illustrate the effect estimate and the weight of each study, along with the overall outcome effect estimate. Heterogeneity can also be inspected on this plot. Clinical heterogeneity can also be noted for various reasons, e.g., discrepancies between the time of assessments and different protein dosages provided.

### 2.6. Reporting Bias Assessment and Certainty Assessment

We could not conduct an analysis for publication bias due to the low number of studies included. We did, however, assess the certainty of evidence using GRADEpro GDT software (version: 25 October 2021) [36] as high, moderate, low, or very low according to five criteria, as follows: (i) consistency of effect, (ii) imprecision, (iii) indirectness, (iv) risk of bias, and (v) publication bias.

## 3. Results

### 3.1. Study Selection

**Figure 1:**
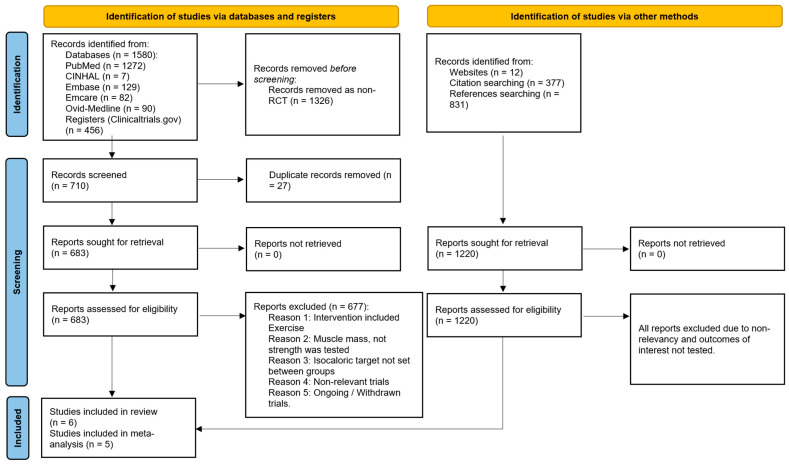
PRISMA 2020 flow diagram for searches of databases, registers, and other sources [30].

The initial database search identified 1580 records (Ovid MEDLINE (90), Embase (129), Emcare (82), CINAHL (7), and PubMed (1272)). An additional 456 records were identified using the online study register (ClinicalTrials.gov) as detailed in Supplementary Table S3. After 1326 non-RCTs were removed, another 27 duplicates were excluded using Zotero (v6.0.12) software. Thereafter, 683 studies remained for title and abstract screening. Additionally, manual searching through online websites, citations, and reference lists of the eligible database-derived records identified a further 1220 records. Of these, six records [15,31,32,33,34,35] met the inclusion criteria, out of which five studies [15,31,32,34,35] were suitable for meta-analysis. All studies included in our meta-analysis [15,31,32,34,35] used HGS as their measure of skeletal muscle strength. Although one trial [33] did fulfil the inclusion criteria for this review, it is not included in the meta-analysis as tidal volume was assumed to be a surrogate measure of respiratory skeletal muscle strength, which differs from the measurement method employed in the other included trials, which is HGS. The PRISMA 2020 flow diagram is shown in Figure 1.

### 3.2. Study Characteristics

Each of the six studies that fulfilled the inclusion criteria [15,31,32,33,34,35] was an RCT. A total of 632 patients were included in the meta-analysis for handgrip strength [15,31,32,34,35]. One study was conducted in China [33]. Two trials were conducted in Australia [15,34]. One trial was completed in Brazil [31]. Another study was conducted in Egypt [35]. Finally, the most recent study was conducted in the Netherlands and Belgium [32].

All of the studies included patients from mixed medical and surgical specialities. In the four studies that reported sepsis, rates varied between 6.6% and 70.7% [15,31,32,33]. All but one study [15] reported the nutritional status of the patients recruited using the NRS-2002 scoring system, the Bedside Subjective Global Assessment (SGA) method, and the NUTRIC scoring system, with each reporting no significant difference between the intervention and control groups [31,32,33,34,35]. Three studies used enteral nutrition (EN) with supplemental parenteral nutrition (PN) if needed [15,31,32]. One study included only EN [33], and two studies provided nutrition exclusively through PN [34,35]. Full details of the study characteristics can be seen in Table 1.

### 3.3. Results of Risk of Bias Assessment

The risk of bias of each study is summarised in Supplementary Table S7. Overall, two trials showed some concerns [32,34], while the rest of the studies were classified as high risk [15,31,33,35]. Studies by Bels et al. and Ferrie et al. had some concerns because of missing outcome measures for some candidates [32,34]. However, both groups were equally affected, and in the Ferrie et al. trial, missing results were imputed using the regression model imputation function in SPSS software version 21. A concern with the study by Fetterplace et al. was the high number of patients who dropped out of the study, with percentages of 80% and 47% in the intervention and control groups, respectively [15]. The study by Azevedo et al. had several issues, including (i) clinicians not being blinded due to the nature of the intervention; (ii) some missing outcome data; and (iii) planned outcome measurements at certain time points not being met for unexplained reasons [31]. A concern regarding the study by Zhang et al. was the significant difference in time from ICU admission to reaching the target protein intake between the control and intervention groups [33]. The study by Youssef et al. [35] had several concerns, including (i) no specified method of randomisation; (ii) missing information on patients’ group allocation; and (iii) no information on the number of participants who underwent an HGS assessment.

### 3.4. Study Results

#### 3.4.1. Skeletal Muscle Strength

**Figure 2:**
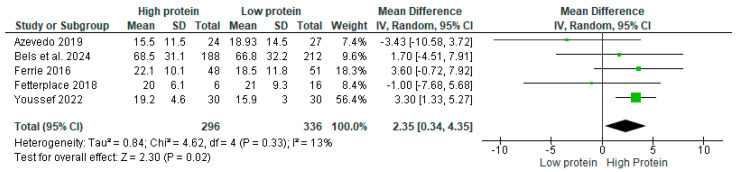
Differences in handgrip strength between the control (low protein) and intervention (high protein) groups [15,31,32,34,35].

The overall mean difference in HGS between the control and intervention groups of the five studies eligible for meta-analysis [15,31,32,34,35] was 2.36 kg (95% CI: 0.37–4.35), with a statistically significant difference between the groups (Figure 2). Statistical heterogeneity between studies, using the I-squared test and *p*-value, was 13% and 0.33, respectively. Hence, based on Cochrane guidelines, heterogeneity is statistically not significant.

#### 3.4.2. Energy and Protein Provision

**Figure 3:**
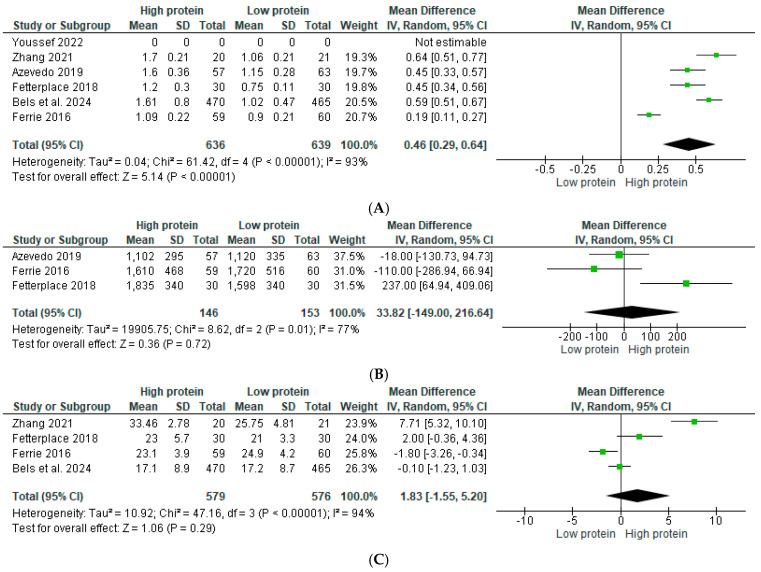
(**A**) Differences in protein provision (g/kg/d) between control (low protein) and intervention (high protein) groups [15,31,32,33,34,35]. (**B**) Differences in total energy provision (kcal/d) between control (low protein) and intervention (high protein) groups [15,31,34]. (**C**) Differences in energy provision relative to body weight (kcal/kg/d) between control (low protein) and intervention (high protein) groups [15,32,33,34].

The overall mean difference in protein provision between control and intervention groups was statistically significant at 0.46 g/kg/d (95% CI: 0.29–0.64) based on the five studies that presented this data [15,31,32,33,34] (Figure 3A). Across these studies, daily energy delivery was presented as both total (kcal/d) and relative (to body weight) (kcal/kg/d) in two studies [15,34], and as relative only in two studies [32,33]. One study presented only total energy provision due to indirect calorimetry being used to guide intake for the intervention group, but a weight-based formula (25 kcal/g/d) was used for the control group [31]. The mean difference in total caloric intake between the control and intervention groups [15,31,34] was 33.82 kcal/d (95% CI: −149.00–216.64) (Figure 3B). However, the mean difference in caloric intake, when calculated on a per kg body weight basis [15,32,33,34] was 1.83 kcal/kg/d (95% CI:−1.55–5.2) (Figure 3C). One study did not provide information about actual protein and caloric intake throughout the study period [35].

Prescribed and delivered proteins and calories are detailed in Supplementary Table S4.

#### 3.4.3. Skeletal Muscle Measurements

Four studies [15,33,34,35] found a significant difference in muscle mass in favour of the intervention groups. Fetterplace et al. reported less quadricep muscle wasting at discharge day with a mean difference (MD) of 0.22 cm (*p* = 0.01) [15]. Studies by Ferrie et al. and Youssef et al. reported that the total volume of three muscles (forearm, biceps, and thigh) was significantly increased in the higher protein group at study day 7, with MD values of 0.5 (*p* = 0.001) and 0.9 cm (*p* = 0.02), respectively [34,35]. One study’s [33] results showed a considerable reduction in diaphragmatic atrophy on CT scan in the first week, with percentage reduction values of 7.14% and 17% (*p* = 0.046) in higher and lower protein groups, respectively.

#### 3.4.4. Functional Outcomes

Five studies [15,31,32,33,34] reported widely varied functional outcomes, which, apart from two studies [32,34], did not show a significant statistical difference. Fetterplace et al. measured physical function using the scored physical function in the intensive care unit test with an MD of −1.1 (*p* = 0.49) [15]. Azevedo et al. used the physical component summary (PCS) scoring system with non-significant differences at 3- and 6-months following ICU discharge, and MDs were 8.4 (*p* = 0.70) and 2 (*p* = 0.93), respectively [31]. Similarly, Zhang et al. did not observe a significant difference between control and intervention groups regarding weaning from mechanical ventilation (MD = 1, *p* = 0.477) [33]. Unlike previously mentioned, Ferrie et al. showed lower Chalder fatigue scores in the intervention group with an MD of −0.8 (*p* = 0.045) [34]. However, intriguingly, in the PRECISe trial, quality of life, as presented by the EuroQoL 5-Dimension 5-level (EQ-5D-5L) score, was lower in the intervention high protein group with a mean difference of –0·05 (95% CI –0·10 to –0·01; *p* = 0·031) [32]. Muscle strength and functional outcomes are detailed in Supplementary Table S8.

#### 3.4.5. Sensitivity Analysis

**Figure 4:**
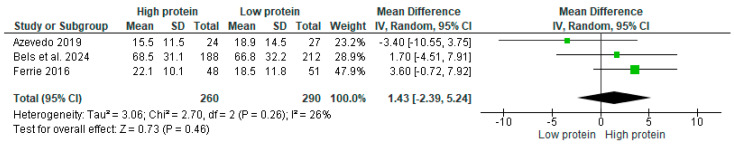
Differences in handgrip strength between control (low protein) and intervention (high protein) groups using sensitivity analysis (isocaloric studies only) [31,32,34].

Sensitivity analysis for HGS was performed on the three studies that reached their isocaloric target between the study groups [31,32,34]. Contrary to the main analysis, there was, however, no significant statistical difference with a mean difference of 1.43 kg (95% CI: −2.39–5.24), which might be due to decreased power (lower number of candidates) and, consequently, a higher level of imprecision (Figure 4). Main and sensitivity analyses are detailed in Supplementary Table S5.

### 3.5. Certainty of Evidence

The certainty of evidence surrounding the impact of the assessed intervention on skeletal muscle strength was graded as low due to serious concerns in the quality assessment of the included studies and a degree of imprecision. However, indirectness and inconsistency had more favourable grades. The grading system of the outcome of interest is shown in Supplementary Table S2.

## 4. Discussion

The aim of this systematic review was to determine the effect of different doses of protein provision on the skeletal muscle function of ICU patients, while maintaining an isocaloric state. Handgrip dynamometry was used to assess the outcome of interest—skeletal muscle strength—across the five studies included in the meta-analysis presented herein [15,31,32,34,35]. A sixth study was identified that fulfilled the original inclusion criteria; however, that study did not measure HGS. Tidal volume and weaning from mechanical ventilation are considered proxies of respiratory muscle strength [33] and, as such, were not included in the meta-analysis. This study concluded that whilst diaphragmatic muscle atrophy was ameliorated with high protein nutrition, no difference was observed in the clinical indices, including weaning and ICU stay duration. Of the studies that did report HGS, one reported this at ICU discharge [31]; two studies [34,35] reported it at various time points during the ICU stay; one reported the best HGS during the ICU stay [15]; and in the most recent one, HGS was assessed at multiple instances following ICU discharge [32]. Irrespective of the timing of assessment, we found that, in line with current nutritional recommendations advocating higher protein provision for ICU patients [13,37], and experimental evidence suggesting higher protein provision can mitigate losses of muscle mass in ICU patients [10,11,38], higher protein provision improved skeletal muscle strength in the ICU population examined in the main analysis of this review.

Of the included studies, only Azevedo et al., Bels et al., and Zhang et al. [31,32,33] managed to deliver the dose of protein currently recommended by European and American guidelines for parenteral and enteral nutrition in critical illness [39,40], with these studies achieving a range of 1.7–1.9 g/kg/d. The studies by Ferrie et al. and Fetterplace et al. [15,34] only provided 1.1 and 1.2 g/kg/d, respectively, in the ‘high-protein’ group, perhaps offering a potential explanation as to why a lack of effect was seen in the Fetterplace study. It is somewhat curious that an improvement in HGS was seen in the study by Ferrie and colleagues, especially given that the mean difference between the two groups was small in this study. Previous studies by Nakamura et al. and Badjatia et al. have shown that 1.5 g/kg/d of protein with adjuvant exercise was needed to improve muscle volume in ICU patients [23,41], and it may be that this intervention is needed to improve muscle function. Despite this, the protein dose across the control/low-protein groups of the five studies included in the meta-analysis ranged from 0.75 to 1.19 g/kg/d [15,31,32,33,34], such that there was an overall significant difference in protein provision between the groups.

While Azevedo’s and PRECISe trials [31,32] reached their isocaloric target, Ferrie et al. [34] provided more calories to their control group. Consequently, these studies were able to test the sole effect of high protein dosage—without enhanced energy—on muscle strength. On the contrary, Fetterplace et al. and Zhang et al. [15,33] did not achieve their isocaloric target, with each providing more calories to the intervention group, which may have impacted their findings. In support of this, higher energy provision during ICU stays has been shown to worsen clinical outcomes, e.g., mechanical ventilation duration and hospital mortality [42]. Furthermore, it may also have had a similar impact on muscle outcomes in a study that tested high-protein nutrition on muscle mass, with no observed benefit [10]. Despite three of the five studies not delivering isocaloric intake between the groups, and one study not reporting the energy supplied to each group [35], overall, neither absolute nor relative (to body weight) energy provision differed between the groups.

Apart from HGS, the included studies also looked at other structural and functional outcomes, including, for example, skeletal muscle thickness, quality of life, and overall physical function. Zhang et al. [33], supporting a beneficial effect of high protein for ICU patients, found that diaphragmatic atrophy, assessed by CT, was lower in the high-protein group around the fifth week post-ICU admission. Similarly, Fetterplace et al. [15] found that high-protein intake led to less quadriceps muscle atrophy (assessed by US) at the point of discharge. Ferrie et al. [34] also used ultrasound and found that the total volume of the three muscle sites (biceps, thigh, and forearm) was significantly higher in the high protein group at study day 7. Notably, this study also reported that an HGS of less than half of its expected value at ICU discharge was correlated with 6-month mortality [34], highlighting the importance of muscle function for these patients. Youssef et al. also found that muscle thickness and nitrogen balance (reflective of protein anabolic status) were higher in the high protein group at study day 7 [35]. Although no linear relationship is observed between muscle mass and functional outcomes, it was previously observed that maintenance of muscle volume in the trajectory of critical illness was correlated with enhanced physical recovery [41]. Referring to alternative functional outcomes (beyond HGS), Azevedo et al. [31] assessed the physical component summary (PCS) of the SF-36 health survey at three and six months post-discharge and found no significant difference between the high- and low-protein groups. Similarly, Fetterplace et al. [15] and Zhang et al. [33] did not show a significant difference between the groups while assessing MRC and weaning from mechanical ventilation, respectively. However, Ferrie et al. [34] observed lower fatigue scores in favour of the intervention group. Finally, only one study included in this review specifically assessed the impact of protein dose on quality of life (QoL), which surprisingly showed a negative outcome in the intervention group, and the potential impact of both muscle mass and function on this is well-reported. For example, Herridge et al. and Fan et al. each reported that ICU-acquired muscle weakness and fatigue had a negative impact on QoL [43,44]. In relation to ICU nutrition, Ridley et al. found no beneficial influence of supplemental parenteral nutrition on different functional outcomes, including QoL [38] in ICU survivors.

To our knowledge, this systematic analysis is the first to concentrate mainly on the influence of different protein doses on skeletal muscle strength in ICU patients. A recently published (2021) systematic review exploring the effect of higher versus lower protein provision for ICU patients on clinical and patient-centred outcomes [45] concluded that no association exists between higher protein nutrition and improved muscle and clinical outcomes. From the 19 studies included in this review, 11 provided information on nutrition delivery relative to body weight, with higher vs. lower protein equating to 1.3 ± 0.48 vs. 0.90 ± 0.30 g/kg/day. Sub-analysis of only two studies in this prior review (both of which are included in this review [15,34]) showed no significant difference in HGS between the high- and low-protein groups, contradicting the main finding of our review. However, the focus of this prior review by Lee et al. [45] (on clinical outcomes) represents the predominance of these endpoints in the literature, with a number of systematic reviews exploring the effect of higher protein doses on these. One such review included six RCTs, of which only one trial tested functional outcomes, including muscle strength, with the others focused on conventional clinical indices such as mortality and length of ICU stay [46]. This review concluded that higher protein nutrition had no positive impact on mortality or hospital/ICU admissions; however, protein provision was at a lower level according to the current recommendations. Another systematic review also investigated six studies—two RCTs and four observational trials—which tested the relationship between energy and/or protein nutrition and skeletal muscle mass and total body protein, and found no association [47]. Skeletal muscle strength was assessed in relation to different nutritional strategies in a recently published review [22], which concluded that there was limited evidence of the effect of different nutritional interventions on physical function. However, the focus of this review was protein timing and delivery route (e.g., early PN and early EN) and not protein dosage.

Purposefully excluded from inclusion in this review, a number of studies have been conducted in the ICU to assess both skeletal muscle mass and functional outcomes in response to high-protein intake as part of a multi-component intervention, most commonly with adjuvant exercise. For example, Nakamura et al. found that thigh muscle mass was better maintained with high protein (1.8 vs. 0.9 g/kg/d), but only if combined with early rehabilitation in the form of electrical muscle stimulation. However, irrespective of rehabilitation, no significant difference was detected in any functional outcome [23]. Furthermore, Badjatia et al. found that high-protein intake (1.8 vs. 1.2 g/kg/d) along with neuromuscular electrical stimulation (NMES) was associated with decreased quadriceps muscle wasting in patients with subarachnoid haemorrhage [41].

Returning to trials of high protein as an independent intervention, a number of studies have looked at the impact on muscle mass, perhaps due to the ease of assessing this (compared to function) in the ICU setting. Despite the relative ease of measurement, these studies have yielded varying results. For example, a single-centre RCT did not find a significant difference in quadriceps muscle thickness between high- and low-protein groups (nor in any of their secondary outcomes such as ICU length of stay, mechanical ventilation duration, indication of renal replacement, incidence of infection, and mortality rates) [10]. Conversely, both Ferrie et al. and Fetterplace et al. (both studies included in this review) found that higher protein provision was associated with attenuation of muscle mass losses [15,34]. This disparity between findings for muscle mass and functional improvements with higher protein (i.e., as seen in Fetterplace et al. [15]) may be due to the complex relationship between muscle mass and function (i.e., factors such as atrophy vs. hypoplasia and the role of neuromuscular input also need to be considered [48], or simply the anatomical disparity between measurement sites (e.g., upper body HGS vs. leg mass). HGS has, however, been shown in numerous clinical cohorts [28,49], including ICU patients [27], to reflect whole-body physical function.

We accept that there are a number of limitations in the present review, both in terms of the small number of eligible studies, which we contend represents a sparsity of research in this space, and methodological considerations with each of these. Firstly, many of the included studies had a high dropout rate and/or could not reach target recruitment numbers. It is worth mentioning that while one trial [34] had processed the missing data by imputation, the other five trials only included the tested data. Secondly, the isocaloric target could not be reached in two of the studies [15,33], and protein targets were not achieved in five of the studies [15,31,32,33,34]. Briefly, delivering different protein doses in the ICU without appropriate countermeasures would impact caloric intake; therefore, it is difficult to reach an isocaloric target whilst delivering different protein doses. However, the overall mean difference of both absolute and relative caloric differences between intervention and control groups across the studies is not significant, which supports the results of this meta-analysis. Furthermore, given the complexity of patient recruitment/retention and the challenges of providing specific nutritional targets in ICU settings, it is not uncommon for such issues to arise in this area of research. Thirdly, heterogeneity in provided protein doses (a range of 1.09–1.7 g/kg/d for the intervention groups across the involved studies) and assessment timepoints (e.g., ICU discharge, following ICU discharge, and at various points during ICU stay) were further matters of concern. Fourthly, the chosen limb was not reported in two studies [32,42]. Two studies [45,46] reported this being conducted in the right arm, while one trial [22] used both arms. However, the same method was used for both groups in each study. It is worth mentioning that although the included studies did not reach the prespecified targets of protein doses, there was a significant statistical difference between both groups in all studies. Furthermore, differences in protein quality between studies need to be recognised as a potentially confounding factor. There was also wide variation between studies regarding the route of administration; some studies used exclusive PN, others used mixed routes when needed, and one study included only EN. Finally, low certainty of evidence and high risk of bias across the studies also added to the limiting factors of this review. We recommend that future research endeavours in this area address these limitations to facilitate the attainment of more definitive conclusions.

In conclusion, according to the studies included in this review, a significant relationship was detected between higher protein provision and skeletal muscle strength in ICU patients. There is, however, a paucity of data to draw firm conclusions on this. Rigorous future research is needed to explore the efficacy of recently recommended higher protein doses in ICU (as recommended by current expert consensus) on muscle function, in the context of an isocaloric state (as a confounding variable). Not only will these studies provide further knowledge in this space, but they will also support evidence around the safety of higher protein in the ICU, which is still in question.

## Figures and Tables

**Table 1:** Patient characteristics for included studies. Data presented as mean (SD), percentage, median (IQR) in Ferrie et al., 2016 [34], and median (min–max) in Youssef et al., 2022 [35]. Abbreviations: APACHE II, Acute Physiology and Chronic Health Evaluation II; NRS, Nutritional Risk Screening; BMI, body mass index; Interv, Intervention Group.

	Age	Sex	APACHE II	Sepsis	Mechanical Ventilation	Nutritional Status (NRS-2002)	Weight (kg) & BMI (kg/m^2^)	Proteins Delivered
Ferrie et al., 2016 [34]	Interv: 67.0 (55.5–74.3)	Interv: M 38 (63%)	Interv: 25.5 (9.4)	Not reported	Interv: 56 (95%)	Interv: 4.3 (1.3)	Interv: 73.2 (16.1)	Intervention group: First 3 study days: 76 (25) g/d 1.17 (0.21) g/kg/d First 7 study days: 76 (26) g/d 1.09 (0.22) g/kg/d
	Control: 64.5 (49.3–70.0)	Control: M 36 (60%)	Control: 23.7 (8.1)		Control: 59 (98%)	Control: 3.9 (1.3)	Control: 77.7 (21.7)	Control group: First 3 study days: 55 (20) g/d 0.87 (1.17)g/kg/d First 7 study days: 60 (21) g/d 0.90 (0.21) g/kg/d
Fetterplace et al., 2018 [15]	Interv: 55 (13)	Interv: M 23 (77%)	Interv: 22 (6.2)	Interv: 3 (10%)	Interv: 30 (100%)	Not reported	Interv: 30 (7.1)	Intervention group: 94 (27) g/d 1.2 (0.3) g/kg/d
	Control: 57 (16)	Control: M 21 (70%)	Control: 20 (5.9)	Control: 1 (3.3%)	Control: 30 (100%)		Control: 29 (5.3)	Control group: 58 (12) g/d 0.75 (0.11) g/kg/d
Azevedo et al., 2019 [31]	Interv: 65.0 (18.8)	Interv: F 23 (40.3%)	Interv: APACHE IV: 81.1 (32.4)	Interv: 12 (21.0%)	Interv: 57(100%)	Interv: 3.9 (0.9)	Not reported	Intervention group: 1.69 (1.33–1.80) g/kg/d
	Control: 67.4 (18.9)	Control: F 31 (49.2%)	Control: APACHE IV: 77.2 (30.7)	Control: 15 (23.8%)	Control: 63 (100%)	Control: 4.1 (1.0)		Control group: 1.13 (0.97–1.34) g/kg/d
Qian Zhang et al., 2021 [33]	Interv: 64.45 (16.17)	Interv: M 12 (60%)	Interv: 21.75 (7.15)	Interv: 13 (65%)	Interv: 20 (100%)	Interv: 4.80 (1.61)	Interv: 22.18 (3.87)	Intervention group: 1.7 (0.21) g/kg/d
	Control: 69.24 (18.15)	Control: M 18 (85.7%)	Control: 20.48 (6.97)	Control: 16 (76.2%)	Control: 21 (100%)	Control: 5.10 (1.58)	Control: 22.84 (4.41)	Control group: 1.06 (0.21) g/kg/d
Youssef et al., 2022 [35]	Interv: 55.5 (33–67)	Interv: M 20 (66.7%) F 10 (33.3%)	Interv: 22 (11–38)	Not reported	Not reported	Interv: SGA(A) 22 (73.3%) SGA(B) 7 (23.3%) SGA(C) 1 (3.3%)	Interv: 89 (84–95)	Intervention group: Not reported
	Control: 48 (40–65)	Control: M 16 (53.3%) F 14 (46.7%)	Control: 21 (10–28)			Control: SGA(A) 18 (60%) SGA(B) 10 (33.3%) SGA(C) 2 (6.7%)	Control: 87.5 (83–90)	Control group: Not reported
Bels et al., 2024 (PRECISe) [32]	Interv: 62 (14)	Interv: M 291 (62%)	Interv: 21 (7)	Interv: 230 (49%)	Not reported	Interv: 4 (1)	Interv: 28 (6)	Intervention group: 1.87 (0.96–2.00) g/kg/d
	Control: 63 (14)	Control: M 309 (67%)	Control: 22 (7)	Control: 229 (49%)		Control: 4 (1)	Control: 27 (5)	Control group: 1.19 (0.63–1.26) g/kg/d

## Supplementary Materials

The following supporting information can be downloaded at https://www.mdpi.com/article/10.3390/nu17162613/s1. Supplementary Material S1: Search strategy and data extraction. Supplementary Table S2: Grading system of the outcome of interest. Supplementary Table S3: Relevant studies in clinical registry. Supplementary Table S4: Prescribed and delivered proteins and calories. Supplementary Table S5: Main and sensitivity analysis. Supplementary Tablel S6: List of studies retrieved from websites. Supplementary Table S7: Risk of bias assessment. Supplementary Table S8: muscle strength and functional outcomes.
