# Supplementary material for: The Effects of Protein Nutrition on Muscle Function in Critical Illness: A Systematic Review and Meta-Analysis
Source: Nutrients. 2025 Aug 12;17(16):2613. doi: 10.3390/nu17162613 (PMC12389481; doi:10.3390/nu17162613)
Supplement: Supplementary file 1 [file nutrients-17-02613-s001.zip › nutrients-3765650-supplementary.pdf]

Supplementary Material S1: Search strategy and data extraction.

Search strings

A sensitive search strategy using exploded MeSH terms was developed. Key search terms were determined by the review question and the inclusion criteria. The search strategy involved developing strings of terms and synonyms to capture three core concepts in the review:

- Concept 1: intervention e.g., protein nutrition.
- Concept 2: population e.g., critically ill patient.
- Concept 3: outcome e.g., muscle / handgrip strength.

These concepts were combined in searches as follows: Concept 1 and Concept 2 and Concept 3.

Keywords

- Intervention keywords
  - Protein nutrition.
  - Protein provision.
  - Dietary protein.
  - Protein supplement.
  - Amino acid.
  - Amino acid infusion.
- Population keywords
  - ICU.
  - ITU.
  - Critical care.
  - Intensive care.
  - Critically ill patient.
  - Severely ill patient.
  - Unstable patient.
- Outcome keywords
  - Muscle strength.
  - Handgrip strength.
  - 6-minute walk test.
  - Physical performance.
  - Physical activity.

- Exploded terms
  - Exp dietary protein.
  - Exp critical care.
  - Exp muscle strength.

Databases

- Ovid Medline.
- Emcare.
- Embase.
- CINHAL.
- Pubmed.

Other search sources

- Study registers (to identify ongoing and unpublished relevant trials)
  1- Clinicalrials.gov
- Snowball searching via references of eligible trials and through citations.
- Websites search (criticalcarenutrition.com).

Data extraction

Data were extracted by reviewers independently via examining titles, abstracts. Full manuscripts were reviewed if closely relevant. All non-RCT studies were excluded.

	Risk of bias	Inconsistency	Indirectness	Imprecision	Publication bias	Large effect	Plausible confounding	Dose response gradient
Skeletal muscle strength	Very serious	Not serious	Not serious	Serious	Undetected	No	No	No

Supplementary Table S2: Grading system of the outcome of interest.

Supplementary Table S3: Relevant studies in clinical registry.

	Trial	Status	Location	Registration ID	Clinical registry
1	**Value of Early Post Operative Amino Acids in Critically Ill Obstetrics on Recovery Enhancement**	Recruiting (last updated February 2025)	Ain-Shams University	**NCT06164301**	Clinicaltrials.gov.uk
2	**Evaluation of Diaphragmatic Function and Quadriceps Muscle Thickness in Patients Receiving High Protein Nutrition**	Not yet recruiting (last updated September 2023)	Zagazig University	**NCT06025760**	Clinicaltrials.gov.uk
3	**Efficacy and Safety of Administration of High Levels of Protein to Critically Ill Patients. (FISIO)**	Active, not recruiting (last updated June 2024)	Different universities in Spain	**NCT05918757**	Clinicaltrials.gov.uk
4	**Replacing Protein Via Enteral Nutrition in Critically Ill Patients (REPLENISH)**	Recruiting (last updated May 2024)	King Abdullah International Medical Research Center	**NCT04475666**	Clinicaltrials.gov.uk
5	**The Effect of Higher Protein Dosing in Critically Ill Patients: A Multicenter Randomized Trial (EFFORTcombo)**	Withdrawn (last updated October 2022)	RWTH Aachen University	**NCT04012333**	Clinicaltrials.gov.uk

Supplementary Table S4: Prescribed and delivered proteins and calories.

	Prescribed proteins	Prescribed calories	Calories delivered	Proteins delivered	Nutrition form
Ferrie 2015	**Intervention group:**<br>1.2 g/kg/d.<br><br>**Control group:** 0.8 g/kg/d.	**Intervention group:**<br>24 Kcal/kg/d.<br><br>**Control group**: 26 Kcal/kg/d.	**Intervention group:**<br>First 3 study days:<br>1053(450) kcal/d<br>23.5(3.9) kcal/kg/d<br><br>First 7 study days:<br>161(468) kcal/d<br>23.1(3.9) kcal/kg/d<br><br>**Control group:**<br>First 3 study days:<br>1700(524) kcal/d<br>26.0(3.8) kcal/kg/d<br><br>First 7 study days:<br>1720(516) kcal/d<br>24.9(4.2)kcal/kg/d	**Intervention group:**<br>First 3 study days:<br>76(25) g/d<br>1.17(0.21) g/kg/d<br><br>First 7 study days:<br>76(26) g/d<br>1.09(0.22) g/kg/d<br><br>**Control group:**<br>First 3 study days:<br>55(20) g/d<br>0.87(1.17)g/kg/d<br><br>First 7 study days:<br>60(21) g/d<br>0.90(0.21) g/kg/d	**PN**<br>- Energy and protein from any EN commenced before randomization.<br>- calories from dextrose solutions or propofol infusions<br>- Content of oral intake was not included in the totals
Fetterplace 2018	**Intervention group**: 1.5 g/kg/d.<br><br>**Control group:** 1 g/kg/d.	**Intervention group:** 25 Kcal/kg/d.	**Intervention group (inc. non-nutritional energy):**<br>1835(340) kcal/d<br>23(5.7) kcal/kg/d	**Intervention group:**<br>94(27) g/d<br>1.2(0.3) g/kg/d	**EN and/or PN and/or protein supplements**<br>- calories from dextrose solutions or

		**Control group:** 25 Kcal/kg/d.	**Control group (inc. non-nutritional energy):**<br>1598(340) kcal/d<br>21(3.3) kcal/kg/d	**Control group:**<br>58(12) g/d<br>0.75(0.11) g/kg/d	propofol infusions
Azevedo 2019	**Intervention group**:<br>2.0-2.2 g/kg/d<br>**Control group:**<br>1.4-1.5 g/kg/d	**Intervention group**:<br>IC daily for the first 3 days, then IC every 2 days until day 10<br>**Control group:**<br>25 kcal/kg/d	**Intervention group**:<br>1139 (890-1278) kcal/d<br>**Control group:**<br>1140 (889-1331) kcal/d	**Intervention group**:<br>1.69 (1.33-1.80) g/kg/d<br>**Control group:**<br>1.13 (0.97-1.34) g/kg/d	**Early EN**<br>- Postpyloric nutrition catheter considered if persistent high aspirates.<br>- SPN if not achieving the target.<br>- TPN if EN contraindicated
Qian Zhang 2021	**Intervention group**:<br>2 g/kg/d.<br>**Control group:**<br>1.2 g/kg/d.	**Intervention group**:<br>25-30 Kcal/kg/d.<br>**Control group:**<br>25-30 Kcal/kg/d.	**Intervention group**:<br>33.46(2.78) Kcal/kg/d.<br>**Control group:**<br>25.75(4.81) Kcal/kg/d.	**Intervention group**:<br>1.7(0.21) g/kg/d.<br>**Control group:**<br>1.06(0.21) g/kg/d.	**Exclusive early EN**
Youssef 2022	**Intervention group**:<br>2 g/kg/d.	**Intervention group**:<br>25-30 Kcal/kg/d.	**Intervention group**:<br>N/A<br>**Control group:**	**Intervention group**:<br>N/A	**Exclusive early PN**

	**Control group:**<br>1 g/kg/d.	**Control group:**<br>25-30 Kcal/kg/d.	N/A	**Control group:**<br>N/A	
Bels 2024	**Intervention group**:<br>2 g/kg/d.<br>**Control group:**<br>1.3 g/kg/d.	**Intervention group**:<br>25 Kcal/kg/d.<br>**Control group:**<br>25 Kcal/kg/d.	**Intervention group**:<br>17 (8.9) Kcal/kg/d.<br>**Control group:**<br>17.2 (8.7) Kcal/kg/d.	**Intervention group**:<br>1.87 (0·96–2·00) g/kg/d.<br>**Control group:**<br>1.19 (0·63–1·26) g/kg/d.	**Initial gradual increase of EN. PN was permitted only after day 8 of admission.**

Supplementary Table S5: Main and sensitivity analysis.

	Number of trials	Number of patients	Heterogeneity	Outcome measure – SMD/MD (95% CI)	Overall effect
Main analysis	Five trials (Azevedo, Ferrie, Fetterplace, PRECISe and Youssef)	Intervention group: 296 patients<br>Control group: 336 patients	I2 = 13%<br>Tau2 = 0.81<br>Chi2 = 4.6	MD = 2.36<br>95% CI = (0.37 – 4.35)	P = 0.02<br>Z = 2.33
Sensitivity analysis	Three trials (Azevedo, PRECISe and Ferrie)	Intervention group: 260 patients<br>Control group: 290 patients	I2 = 26%<br>Tau2 = 3.06<br>Chi2 = 2.70	MD = 1.43<br>95% CI = (-2.39 – 5.24)	P = 0.46<br>Z = 0.73

Supplementary Table S6: List of studies retrieved from websites.

	Studies
1	The RE-ENERGIZE Trial: A RandomizEd Trial of ENtERal Glutamine to minimIZE Thermal Injury
2	PEP uP Protocol in Surgical Patients: A PEP uP RCT
3	PERFormance Enhancement of the Canadian nutrition guidelines by a Tailored Implementation Strategy: The PERFECTIS Study
4	Validation of Bedside Ultrasound of Muscle Layer Thickness of the Quadriceps in the Critically Ill Patient: The VALIDUM Study
5	Promotion of Regular Oesophageal motility to Prevent regurgitation and Enhance nutrition intake in Long-stay ICU patients. A multicenter, Phase II, sham-controlled, randomized trial: The PROPEL Study
6	A Randomized Trial of Glutamine and Antioxidants in Critically Ill Patients
7	Improving the Practice of Nutrition Therapy in the Critically ill: An International Quality Improvement Project
8	A Randomized Trial Of Supplemental Parenteral Nutrition in Under and Overweight Critically Ill Patients: The TOP UP Trial
9	LOVIT (lessening organ dysfunction with vitamin C)
10	The NUTRIATE Study: Nutritional adequacy therapeutic enhancement in the critically ill: A randomized double blind, placebo-controlled, active-compared trial of the motilin receptor agonist GSK962040
11	SodiUm SeleniTe Administration IN Cardiac Surgery (SUSTAIN CSX®-trial):A multicentre randomized controlled trial of high dose sodium-selenite administration in high risk cardiac surgical patients

Supplementary Table S7: Risk of bias assessment.

	**Randomization process**	**Effect of assignment to intervention**	**Missing outcome data**	**Outcome measurement**	**Reported results selection**	**Overall**
Ferrie et al., 2015	Low risk	Low risk	Some concerns (>10% missing outcome data from both groups, but imputation was possible).	Low risk	Low risk	Some concerns
Fetterplace et al., 2018	Low risk	Low risk	High risk (>10% missing outcome data from both groups and uneven across groups).	Low risk	Low risk	High risk
Azevedo et al., 2019	Low risk	High risk (not blinded)	High risk (>10% missing outcome and even across both groups)	Some concerns (outcome assessment was not properly blinded)	Some concerns (protocol on clinical trials registry, shows outcome measurements planned for different time points)	High risk
Qian Zhang et al., 2021	Some concerns (allocation concealment not mentioned)	Low risk	Low risk	Some concerns (assessors were not blinded)	Some concerns (no available protocol)	High risk
Youssef et al., 2022	Some concerns (method of randomization not mentioned)	High risk (no information available to estimate the effect of assignment)	High risk (no information about the number of patients who underwent assessment for the outcome of interest)	Low risk	Some concerns (no available protocol)	High risk
Bels et al., 2024	Low risk	Low risk	Some concerns (>10% missing outcome data from both groups)	Low risk	Low risk	Some concerns

Supplementary Table S8: muscle strength and functional outcomes.

	Azevedo et al 2019	Fetterplace et el (FEED trial) 2018	Ferrie et al 2015	Youssef et al 2022	Bels et al (PRECISE) 2024	Qian Zhang et al 2021
HGS outcomes	No significant difference between groups	No significant difference between groups	Favourable outcome in the intervention group	Favourable outcome in the intervention group	No significant difference between groups	
Physical function and quality of life instruments used	physical component summary (PCS) scoring system	the scored Physical Function in Intensive Care Unit Test	Chalder fatigue scores	-----	EuroQoL 5-Dimension 5-level (EQ-5D-5L) score	weaning from mechanical ventilation
Physical function and	No significant difference between groups	No significant difference between groups	Favourable outcome in the	-----	worse outcome in the	No significant difference between groups

quality of life outcomes			intervention group		intervention group	
